# Adhesive ginsenoside compound K patches for cartilage tissue regeneration

**DOI:** 10.1093/rb/rbad077

**Published:** 2023-08-31

**Authors:** Jun-Ho Yang, Hyun Ho Shin, Donghyeon Kim, Ji Hyun Ryu, Eun-Jung Jin

**Affiliations:** Department of Biological Sciences, College of Health Sciences, Wonkwang University, Iksan, Jeonbuk 54538, South Korea; Department of Chemical Engineering, Wonkwang University, Iksan, Jeonbuk 54538, South Korea; Department of Biological Sciences, College of Health Sciences, Wonkwang University, Iksan, Jeonbuk 54538, South Korea; Department of Carbon Convergence Engineering, Wonkwang University, Iksan, Jeonbuk 54538, South Korea; Integrated Omics Institute, Wonkwang University, Iksan, Jeonbuk 54538, South Korea; Department of Biological Sciences, College of Health Sciences, Wonkwang University, Iksan, Jeonbuk 54538, South Korea; Integrated Omics Institute, Wonkwang University, Iksan, Jeonbuk 54538, South Korea

**Keywords:** ginsenoside compound K, adhesive patch, drug delivery, hydrocaffeic acid-conjugated chitosan, osteoarthritis

## Abstract

Biomaterial-based drug delivery systems have been developed to expedite cartilage regeneration; however, challenges related to drug recovery, validation, and efficient drug delivery remain. For instance, compound K (CK) is a major metabolite of ginsenosides that is known to protect against joint degeneration by inhibiting the production of inflammatory cytokines and the activation of immune cells. However, its effects on cartilage degradation and tissue regeneration remain unclear. Additionally, tissue-adhesive drug delivery depots that stably adhere to cartilage defects are required for CK delivery. In this study, CK-loaded adhesive patches were reported to seal cartilage defects and deliver CK to defect sites, preventing cartilage degradation and accelerating cartilage tissue regeneration. Adhesive patches are stable and suitable for application in surgical procedures under physiological conditions and show excellent adhesiveness to cartilage surfaces. In addition, there were no significant differences in the adhesive polymeric networks before and after CK loading. CK-loaded hydrocaffeic acid-conjugated chitosan patches significantly inhibited the stimulation of cartilage-degrading enzymes and apoptosis in osteoarthritic cartilage by releasing CK in cartilage defects. Additionally, the NFkB signaling pathway of released CK from the adhesive patches in the treatment of osteoarthritis is revealed. Thus, the CK-loaded adhesive patches are expected to significantly contribute to cartilage regeneration.

## Graphical Abstract

**Figure:**
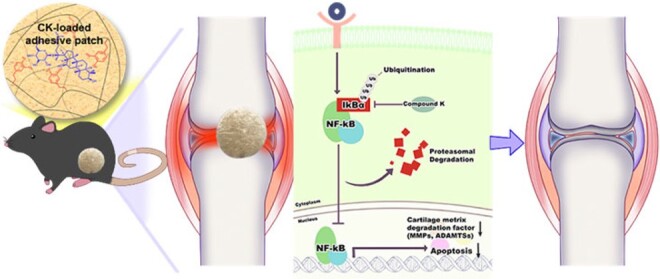

## Introduction

Cartilage regeneration is a promising strategy for the treatment of osteoarthritis (OA), a degenerative joint disease affecting millions of people worldwide [1]. However, regenerated cartilage tissue often has poor properties compared with natural cartilage tissue. Moreover, poor cartilage regeneration could stimulate joint degeneration and worsen symptoms, including pain, stiffness, and loss of mobility [2]. There are several reasons for the poor properties of regenerated cartilage. First, the new tissue may not have the same structure and composition as natural cartilage. For instance, regenerated cartilage may have a different ratio of the cartilage matrix, which may affect its mechanical properties. The second factor is the cartilage characteristics. Cartilage lacks blood vessels and nerves [3], meaning that it has a limited capacity for self-repair and a reduced ability to sense pain or inflammation. Additionally, cartilage has a low cell density and a limited supply of nutrients [4], which can make it difficult to stimulate regeneration. Another factor that affects cartilage regeneration is the location and severity of the injury. The cartilage in weight-bearing joints, such as the knee and hip [5], is prone to damage and may require extensive regenerative effort. To address this challenge, new techniques for improving the properties of regenerated cartilage tissue have been developed using biomaterials or scaffolds to provide structural support for the new tissue or growth factors or other signaling molecules to promote the development of more robust cartilage tissue.

Compound K (CK), a natural compound found in ginseng, possesses anti-inflammatory and antioxidant properties (https://www.gcoopinfo.com/post/the-benefits-of-compound-k-ginseng) [6]. In addition, some studies have suggested that CK has a potential protective effect against rheumatoid arthritis, which may promote cartilage regeneration and protect against further joint degeneration by inhibiting the production of inflammatory cytokines, activation of immune cells such as macrophages, B- and T-cells, and the migration and proliferation of fibroblast-like synoviocytes [7, 8]. Overall, although CK may have potential as a treatment for OA, further research is required to validate its effects.

For the successful delivery of chemical drugs, various biocompatible materials have been developed to increase drug delivery efficiency [9–13]. In addition, tissue-adhesive polymeric materials are useful for delivering drugs to target tissues via various interactions between the tissue-adhesive materials and target tissues [14–16]. Several reactive functional group-containing polymeric materials, including N-hydroxysuccinimide (NHS) esters, cyanoacrylates, aldehydes, catechol, aryl azides, and isocyanates, can covalently bind to tissues and are candidates for tissue adhesives [17]. In particular, mussel-inspired catechol-containing polymers (i.e. chitosan-catechol [18–20], hyaluronic acid-catechol [21, 22], and alginate-catechol [23, 24]) have been extensively synthesized as tissue-adhesive materials for hemostasis, tissue repair, antimicrobial, drug delivery, and cell therapy. For instance, dendritic mesoporous organic silica nanoparticle-incorporated catechol-conjugated gelatin and hyaluronic acid hydrogels readily encapsulate dexamethasone, which can achieve controlled release of drugs, resulting in successful chondrogenic differentiation by inducing the activation of HIF-1ɑ [25]. In addition, alginate-catechol-containing composite hydrogels with gelatin and acrylated β-cyclodextrins show excellent properties of injectability, self-healing properties, and tissue adhesion that can be applicable for cartilage tissue engineering [26]. In addition, hydrogels with pulsed electromagnetic fields induce chondrogenesis and cartilage repair in rat osteochondral defect models. Thus, catechol-containing polymeric materials have enormous potential as drug delivery depots and tissue engineering scaffolds for cartilage regeneration.

In this study, we developed CK-loaded adhesive hydrocaffeic acid-conjugated chitosan (CK/CHI-HCA) patches for patients with OA. Previously, we synthesized CHI-HCA gel patches as capping materials for the directional release of growth factors from heparin-conjugated fibrin gels to prevent migration and dispersion of MSCs from osteochondral defects [27]. We hypothesized that the strong tissue adhesiveness of CHI-HCAs with barrier formation would be useful for local delivery of CK to cartilage tissues. As expected, the CK/CHI-HCA patches showed excellent tissue-adhesive properties in both mouse subcutaneous regions and porcine cartilage tissues. Notably, the CK/CHI-HCA patches are suitable for application to both soft and hard tissues. In addition, the CK/CHI-HCA patches significantly reduced the expression levels of MMP-3 and ADAMTS-4, indicating inhibition of the stimulation of cartilage-degrading enzymes and apoptotic cell death in OA cartilage. Our data suggest that the release of CK from the adhesive patches inhibits NFkB signaling, resulting in cartilage tissue regeneration in OA models. Thus, the adhesive CK-loaded patches can be used as adhesive drug depots for cartilage tissue engineering.

## Materials and methods

### Materials

Chitosan (medium-molecular weight), dimethyl sulfoxide (DMSO), and methanol (MeOH) were purchased from Sigma-Aldrich (St. Louis, MO, USA). Hydrocaffeic acid (HCA), N-hydroxysuccinimide (NHS), and 1-ethyl-3-(3-dimethyl aminopropyl) carbodiimide (EDC) were purchased from TCI-SU (Tokyo, Japan). CK was purchased from ChemFaces (Wuhan, China). All the other chemicals were of analytical grade.

### Synthesis of CHI-HCA

CHI-HCAs were synthesized using standard carbodiimide chemistry. Briefly, chitosan (1 g) was dissolved in pH 2 DDW (100 ml), and the pH was adjusted to 5 using 1 N NaOH. After the complete dissolution of chitosan, HCA (1.13 g), EDC (0.96 g), and NHS (0.71 mg) were added to the chitosan solution dropwise. The pH was maintained between 4.5 and 5.5 during the reactions. After 12 h, the product was purified using a dialysis membrane (MWCO: 12–14 kDa, SpectraPor) against a pH 2 NaCl (10 mM) solution for 2 days and DDW for 4 h. CHI-HCA was obtained by lyophilizing the final product. The HCA conjugation of CHI-HCA was confirmed by 1H NMR (Bruker Avance, 500 MHz) and UV-Vis spectrophotometry (UV-1900i, Shimadzu).

### Preparation of CK/CHI-HCA patches

The CHI-HCA patches without CK were prepared using a freeze-drying method. CHI-HCA (0.5, 1, 2, and 4 wt%) was dissolved in DDW and subsequently freeze-dried. The CK/CHI-HCA patches were prepared using a method similar to that described above. Briefly, CHI-HCA (1 wt%) was dissolved in DDW, and CK (5 wt%) was dissolved in MeOH. The HCA solution (0.2 ml) was mixed with the CK solution (20 μl). After homogeneous mixing, the CK/CHI-HCA solution was evaporated to remove MeOH and then freeze-dried.

### Rheological study

The rheological analysis of the CK/CHI-HCA patches in pH 7.4 PBS solution was performed using a rotational rheometer (Kinexus Lab+, Netzsch, Germany). First, CHI-HCA patches without CK prepared with different concentrations of CHI-HCA solution (0.5, 1, 2, and 4 wt%) were placed in the pH 7.4 PBS solution. After 24 h, CHI-HCA hydrogels were obtained. The elastic (*G*′) and viscous (*G*″) moduli were monitored as functions of frequency. The frequency was varied from 0.1 and 10 Hz with 21 points. To compare the CHI-HCA and CK/CHI-HCA hydrogels, a CHI-HCA solution (1 wt%) was used to prepare the CK/CHI-HCA patches. All measurements were performed in triplicate.

### Study on tissue adhesive properties

The tissue adhesive properties of the CK/CHI-HCA patches on mouse subcutaneous regions and porcine cartilage (Bucknam Butcher’s Shop, South Korea) were evaluated by measuring the tensile strength using a universal testing machine (UTM, Instron 5583, Instron, USA) with a 50 N load cell. Before measuring tensile strength, mouse subcutaneous regions were cut into 1×1 cm^2^ pieces and attached to the edges of polyethylene terephthalic acid (PET) films (1×5 cm^2^) using commercially available cyanoacrylate adhesives. After washing the tissues on the PET films, the two tissues were overlapped, and CK/CHI-HCA patches were applied between them. Tensile strength was monitored after pulling the probe at a loading rate of 1 mm/min.

### Cytotoxicity of CK/CHI-HCA extracts

Cell viability was measured using Muse Annexin V and Dead Cell Kits (Luminex). Cells were incubated with annexin V and propidium iodide solutions, and fluorescence was analyzed using a Muse Cell Analyzer (Merck Millipore).

### Immature murine articular chondrocytes culture

The immature murine articular chondrocytes (iMACs) were isolated from postnatal Day 5 pups according to a previously published method (Song et al., 2018). iMACs were cultured with Dulbecco’s modified Eagle’s medium (Gibco) with 10% fetal bovine serum (Gibco) and 100 units/ml of penicillin and streptomycin at 37°C supplied with 5% CO_2_.

### TUNEL assay

In Situ Cell Death Detection Kim (Roche) was used with deparaffinized cartilage sections according to the manufacturer’s instructions. Images were acquired by fluorescence microscopy after nuclear staining with 4′,6-diamidino-2-phenylindole.

### Cell proliferation assay

Cell proliferation was measured using the Quick Cell Proliferation Colorimetric Assay Kit (Biovision, K301) with a Muse Cell Analyzer (Merck Millipore) following the manufacturer’s instructions.

### Experimental animals

Destabilization of the medial meniscus (DMM) surgery was performed on the left knee joint of 8-week-old mice without cutting the ligament as a control. At 8 weeks after DMM surgery, the knee joint tissues were processed for histological analysis. All animal experiments were performed in accordance with the guidelines of the Institutional Animal Care and Use Committee (IACUC) of Wonkwang University. All animal experiments were approved by the Animal Ethics Committee of Wonkwang University (WKU22-122).

### Histological analysis

Cartilage samples were fixed with 10% neutral-buffered formalin for 24 h and decalcified using 0.5 M ethylenediaminetetraacetic acid solution for a week. After paraffin embedding, blocks were cut at 5 μm thickness and stained with safranin O. For immunohistochemical analysis, deparaffinized sections were incubated with primary antibodies overnight at 4°C in a humidified chamber. The sections were subsequently developed using ImmPACT DAB (Vector Laboratories, No. SK‐4105). The following antibodies were used for immunohistochemical analysis: MMP13 (Biovision, No. 3533), MMP 3 (EMD MILLIPORE, No. MAP3306), ADAMTS4 (Abcam, No. AB28285), pIΚB (Cell Signaling, No. 2859), IκB (SANTACRUZ, No. SC-847), and horseradish peroxidase-conjugated goat anti-rabbit IgG (Enzo Life Sciences, No. ADI‐SAB‐300).

### Quantitative real‐time polymerase chain reaction

Reverse-transcribed using 5X All‐in‐One RT Master Mix (ABM, No. G492). Quantitative real‐time polymerase chain reaction (qRT‐PCR) was performed using AMPIGENE qPCR Green Mix (Enzo Life Sciences, No. ENZ‐NUC104‐1000). RN18S was used as an endogenous control. The qRT-PCR primer sequences used in this study are listed in Supplementary Table S1.

## Results and discussion

### Synthesis and preparation of CK/CHI-HCA patches

**Figure 1.**
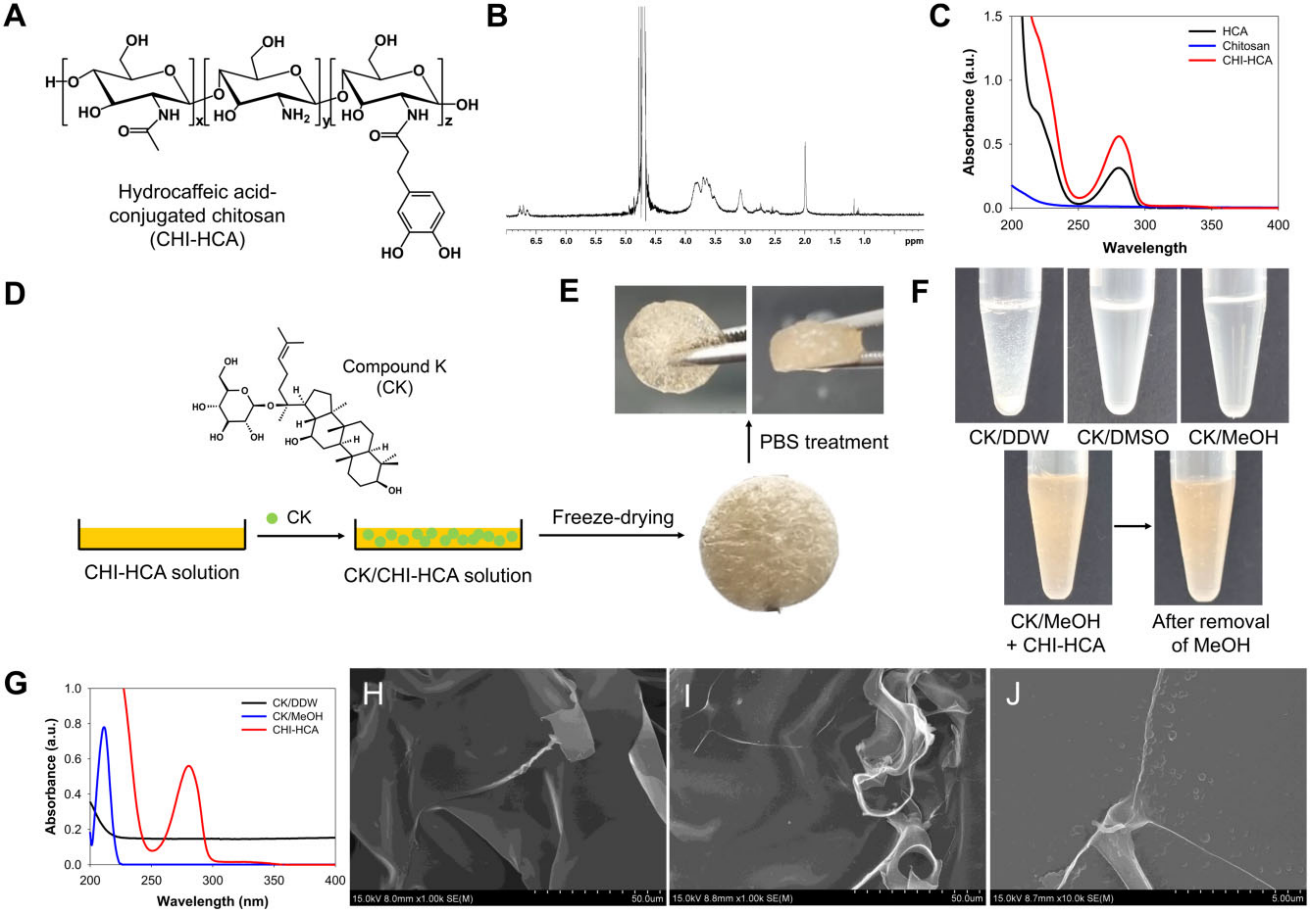
(**A**) Chemical structures, (**B**) ^1^H NMR, and (**C**) UV-Vis spectra of CHI-HCA. (**D**) Schematic illustrations of fabrication of CK/CHI-HCA patches with photographic images. (**E**) Photos of CK/CHI-HCA patches after PBS treatments. (**F**) Photographic images of CK in DDW, CK in DMSO, CK in MeOH, mixture solutions of CK in MeOH and CHI-HCA solution, and after removal of mixture solutions. (**G**) UV-Vis spectra of CK in DDW, CK in MeOH, and mixture solution CK and CHI-HCA. SEM images of CHI-HCA (**H**), CK/CHI-HCA patches (**I**), and CK/CHI-HCA patches with 10k magnifications (**J**).

Hydrocaffeic acid-conjugated chitosan (CHI-HCA) was synthesized using an EDC coupling agent. As shown in Figure 1A, hydrocaffeic acid (HCA) was introduced into the chitosan backbone by forming amide linkages between the carboxylic acid groups of HCA and the amine groups of chitosan. Hydrocaffeic acid conjugation to the chitosan backbone was confirmed by ^1^H NMR spectroscopy, and UV-Vis spectra were obtained. The catechol protons of CHI-HCA were found in the range of 6.5–7.0 ppm in the ^1^H NMR spectra (Figure 1B). In addition, an absorbance peak at 280 nm caused by HCA conjugation was observed in the UV-Vis spectra of CHI-HCA (Figure 1C). The degree of HCA substitution of CHI-HCA was approximately 8.7%, which was calculated using its absorbance at 280 nm with standard curves of HCA concentrations. To prepare the CK-containing CHI-HCA patches, CHI-HCA (1 wt%) was dissolved in DDW, and CK was dissolved in MeOH. After complete dissolution, the CHI-HCA solution was mixed with the CK solution and freeze-dried (Figure 1D). The CK/CHI-HCA patches were free-standing in pH 7.4 PBS solution and handled using forceps (Figure 1E). As shown in Figure 1F (top, first), CK in DDW formed insoluble precipitates because of the lack of solubility of CK in the aqueous solution. Generally, DMSO is used to dissolve CK for therapeutic purposes. MeOH was used as the solvent because it can be readily removed by evaporation. CK in both DMSO and MeOH was clear, indicating complete dissolution (Figure 1F, top, second, and third). In addition, the mixed solutions of CK in MeOH and CHI-HCA in DDW were clear, even after the removal of MeOH at the same concentrations as the CHI-HCA patch preparations (Figure 1F, bottom). CK alone in DDW showed an overall upshift in the UV-Vis spectra at all wavelengths owing to its insolubility. However, CK and CK/CHI-HCA showed no upshift above 350 nm, indicating that both were soluble in DDW. The small shoulder of CK/CHI-HCA at wavelengths of 300–350 nm may be due to the slight oxidation of CHI-HCA during the evaporation of MeOH. Next, we confirmed the morphological changes before and after adding CK to the CHI-HCA patches. As shown in Figure 1H and I, there were no significant differences in the morphologies of CHI-HCA (Figure 1H) and CK/CHI-HCA (Figure 1I). However, nanoparticle-like structures in the CK/CHI-HCA patches were found at 10k magnifications, probably because of the interactions between CK and CHI-HCA.

### Characterizations of CK/CHI-HCA patches

**Figure 2.**
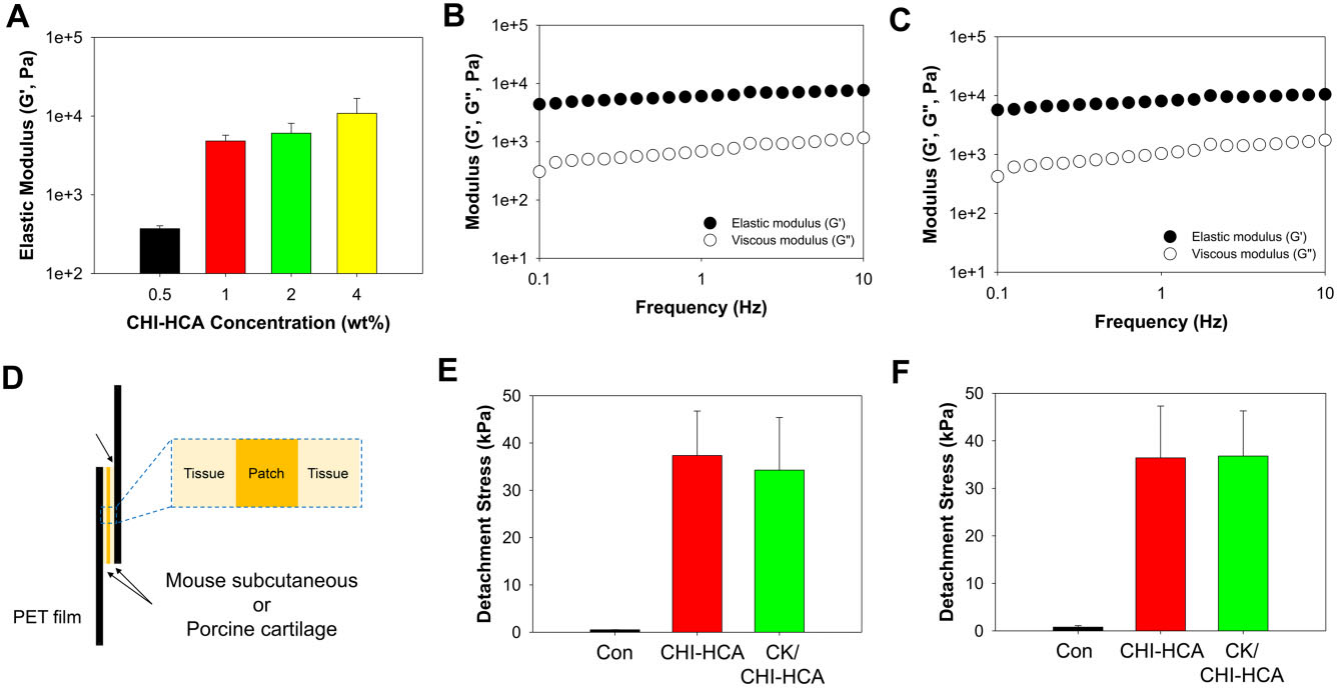
(**A**) Elastic modulus changes of CHI-HCA patches as a function of concentration after treatments of pH 7.4 PBS solutions. Frequency sweep measurements of CHI-HCA (**B**) and CK/CHI-HCA (**C**) hydrogel patches. (**D**) Illustrations of tissue adhesive properties of CK/CHI-C patches. Tissue adhesive properties of CK/CHI-HCA patches on mouse subcutaneous regions (**E**) and porcine intestine (**F**).

We performed rheological analysis of the CHI-HCA patches after treatment with PBS. The elastic modulus values (*G*′) of CHI-HCA patches in the PBS solutions after 24 h were increased as a function of CHI-HCA concentration (Figure 2A). The *G*′ values of CHI-HCA patches were 0.37 ± 0.1 kPa for 0.5 wt%, 4.8 ± 0.9 kPa for 1 wt%, 6.1 ± 2.0 kPa for 2 wt%, and 10.8 ± 5.9 kPa for 4 wt%. We fixed the concentration of CHI-HCA at 1 wt% because the *G*′ values were similar between 1 and 4 wt%. Also, the elastic (*G*′) and viscous (*G*′′) modulus values of CHI-HCA were monitored as a function of frequency (Figure 2B). The *G*′ values were higher than those of the *G*′′ values, and the *G*′ values were sustained as a function of frequency, indicating that the polymer networks were formed by crosslinking of CHI-HCA. Due to the self-oxidative crosslinking of chitosan molecules by catechol groups in an intra- and intermolecular fashion [18], CHI-HCA forms 3D structures without any additives in the pH 7.4 PBS or media. In addition, after the addition of CK, CHI-HCA showed a similar hydrogel behavior in the frequency sweep measurements, which might be due to the low composition of CK (Figure 2C). To verify the adhesive properties of CK/CHI-HCA, we used a modified lap-shear test method by attaching tissues to both sides of each adherend (i.e. PET film) using a UTM. As shown in Figure 2D, the CK/CHI-HCA patches were attached to the tissue surfaces, and the tensile strengths were measured. The detachment stresses of CHI-HCA and CK/CHI-HCA between the mouse subcutaneous regions were 37.3 ± 9.3 and 34.2 ± 11.1 kPa, which are far higher than those of control groups (0.51 ± 0.02 kPa). In addition, the detachment stress of CK/CHI-HCA attaching to the porcine cartilages was 36.8 ± 9.5 kPa, which was similar to the mouse subcutaneous regions.

**Figure 3.**
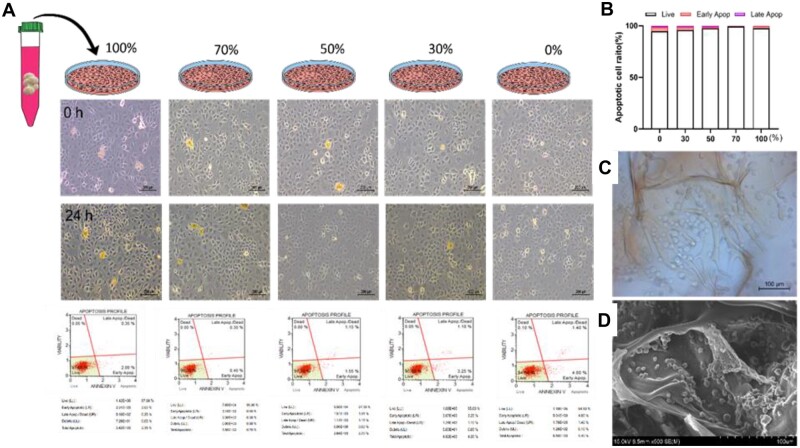
(**A**) Cytotoxicity and apoptosis profiles of extracts of CK/CHI-HCA patches on the iMACs. (**B**) Apoptotic cell ratios of extracts with different concentrations. (**C**) Optical and (**D**) SEM images of cell-laden CK/CHI-HCA patches.

To confirm cytotoxicity, the CHI-HCA patches were soaked in 10 ml of medium for 24 h. After removing the patches, immature murine articular chondrocytes (iMACs) were cultured with different concentrations (30%, 50%, 70%, and 100%) of CHI-HCA extracts for 24 h (Figure 3A). There was no significant difference in cell viability with increasing CHI-HCA extract dilution (Figure 3B), demonstrating that the CHI-HCA patches were not cytotoxic. Optical and SEM images revealed healthy cells inside the CHI-HCA patches (Figure 3C and D).

### Efficacy of CK/CHI-HCA patches in the OA model

**Figure 4.**
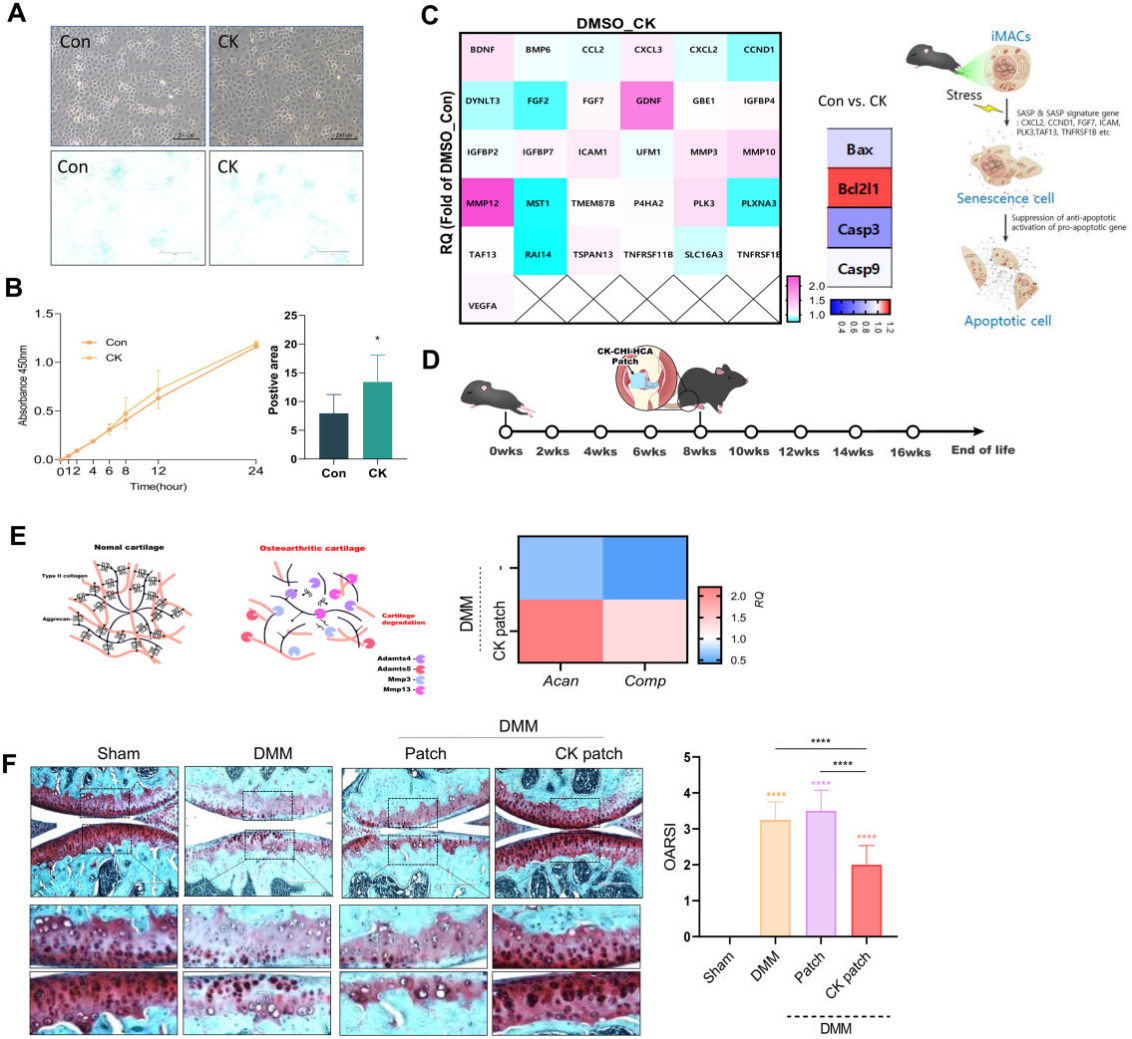
CK effect on iMACs: (**A**) Images of bright field and Alcian blue staining; (**B**) Proliferation assay; (**C**) Expression level of genes in cellular senescence and apoptosis. CK effect on OA pathogenesis: (**D**) Experimental scheme; (**E**) Expression level of cartilage matrix genes; (**F**) Images of Safranin O staining and OARSI score.

iMACs were cultured to explore the effects of CK on chondrocyte differentiation (Figure 4A). Chondrocyte proliferation was significantly altered, whereas chondrogenic differentiation was significantly increased in iMACs treated with CK compared to WT iMACs (Figure 4B). In addition, the exposure of cells to CK decreased the expression of genes involved in cellular senescence and apoptosis (Figure 4C). These data suggest that CK promotes chondrogenic differentiation by regulating cell survival and apoptosis.

**Figure 5.**
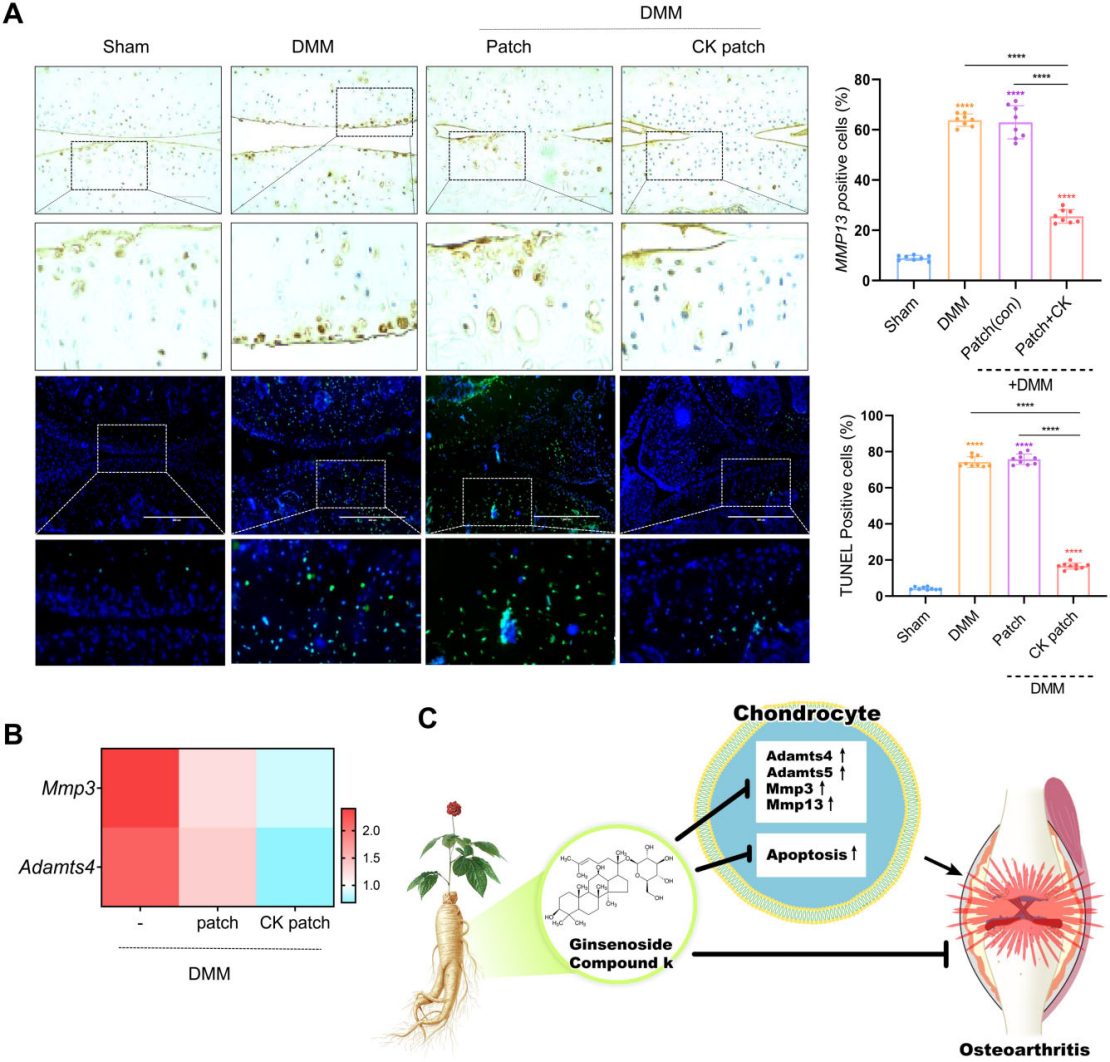
CK effect on OA pathogenesis: (**A**) Expression level of MMP13 and apoptotic cells (left panel) and the percentage of MMP13- and TUNEL cells (right panel); (**B**) Expression level of cartilage degrading enzymes. (**C**) Diagram of the proposed mechanism by which CK suppressed OA pathogenesis via suppression of cartilage degrading enzyme and chondrocyte death.

To investigate the effects of CK on the pathogenesis of OA, DMM was performed to induce OA pathological conditions (Figure 4D). In OA cartilage, degradation of the cartilage matrix, such as aggrecan (Acan) and cartilage oligomeric matrix protein (Comp), due to the action of proteolytic enzymes, is central to OA pathology (Figure 4E). MMP-13 may be the most important in OA because it preferentially degrades type II collagen, and it has also been shown that expression of MMP-13 greatly increases in OA. ADAMTs (a disintegrin and metalloprotease with thrombospondin motifs) belong to a family of extracellular proteases known as the aggrecanase, and ADAMTS-4 and ADAMTS-5 appear to be major enzymes in cartilage degradation in OA. The expression levels of *Acan* and *Comp* were significantly increased in the cartilage with CK-loaded CHI-HCA patches in DMM mice. Severe cartilage degradation, as assessed by safranin O staining, observed in the cartilage of DMM mice was dramatically reduced in cartilages with CK-loaded CHI-HCA patches (Figure 4F). Immunohistochemical analysis showed that the number of MMP13-positive cells, as well as TUNEL-positive cells, was significantly decreased in the cartilage with CK-loaded CHI-HCA patches of DMM mice (Figure 5A). In addition, the expression levels of *MMP-3* and *ADAMTS-4* were significantly reduced in the cartilage with CK-loaded CHI-HCA patches in DMM mice (Figure 5B). These data suggest that the stimulation of cartilage-degrading enzymes and apoptosis in OA cartilage could be inhibited by CK delivered by the CHI-HCA patch into the cartilage.

**Figure 6.**
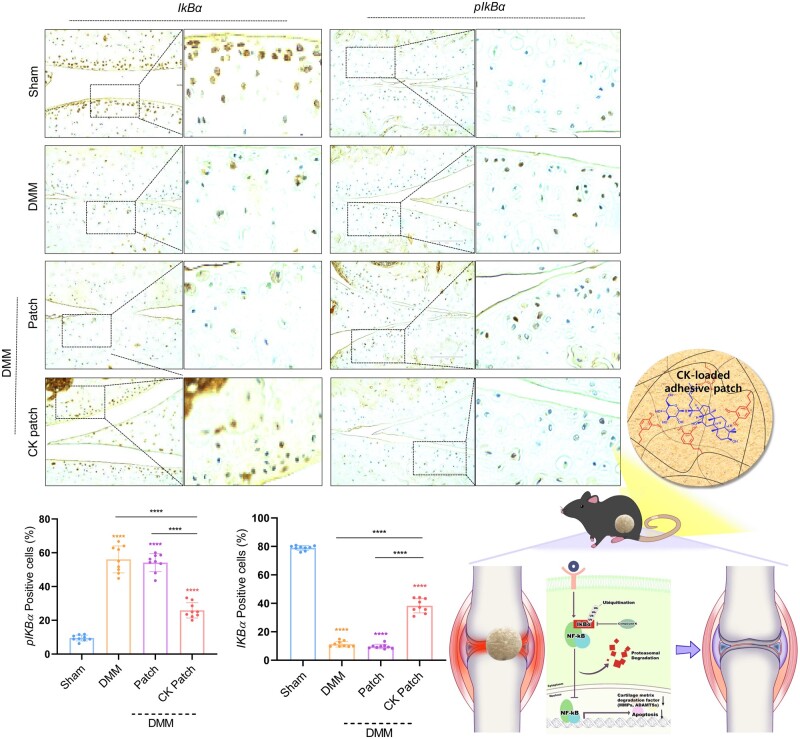
Expression level of IkBα and pIkBα (upper panel) and the percentage of positive cells (lower panel). Diagram of the proposed mechanism by which CK suppressed OA pathogenesis via suppression of cartilage degrading enzyme and chondrocyte death through modulating IkB-NFkB signaling axis.

**Figure 7.**
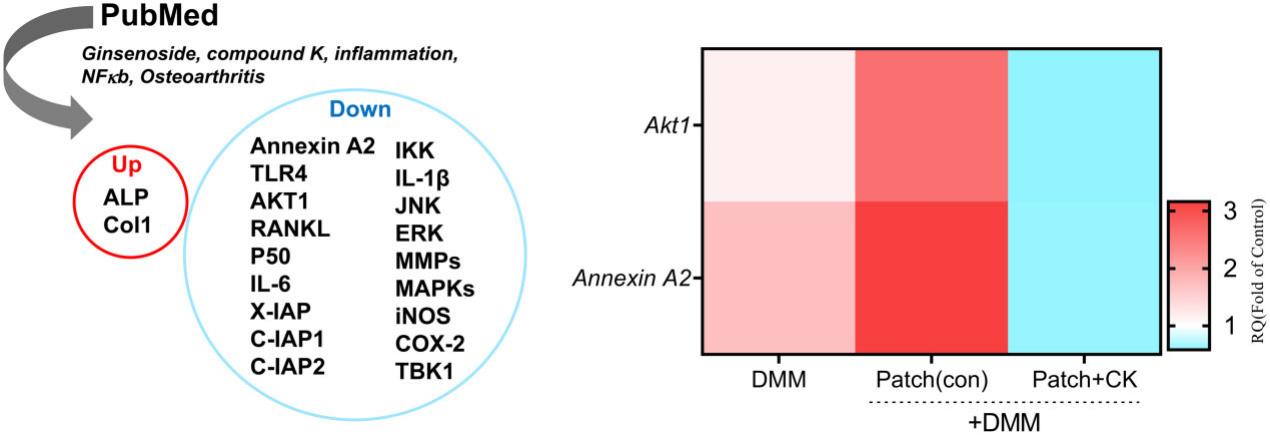
*In silico* analysis using PubMed (left panel) and the expression level of AKT1 and Annexin A2 (right panel).

To identify the underlying signaling mechanism, we searched PubMed and identified common signaling pathways regulated by OA pathogenesis and CK treatment. Among them, we found that the number of pIkBα-positive cells was significantly decreased, whereas the number of IkBα-positive cells was significantly increased in the cartilages with CK-loaded CHI-HCA patches of DMM mice compared to DMM cartilage (Figure 6), suggesting the inhibition of NFκB signaling with CK delivery into cartilage. In addition, among upstream signaling pathway of NFκB searched in PubMed (Figure 7), the expression level of AKT1 and Annexin A2 were significantly decreased in the cartilages with CK-loaded CHI-HCA patches of DMM mice. Akt is known to regulate transcriptional activity of NFκB via degradation of inhibitor of κB [28]. In particular, Annexin A2 has been suggested as a cellular target for CK. CK acts as an inhibitor of Annexin A2, preventing Annexin A2 from binding to p50 and inhibiting NFкB activation [29]. Our data suggested that delivery of CK-loaded CHI-HCA patches inhibits NFкB signaling possibly through modulation of Akt1 or Annexin A2 expression. In summary, delivery of CK by CHI-HCA patch into DMM cartilage suppressed the activation of cartilage-degrading enzymes and apoptotic cell death, possibly via inhibiting NFkB signaling.

## Conclusion

In summary, ginsenoside CK-loaded adhesive patches were developed for cartilage tissue engineering. The CHI-HCA patches showed excellent adhesion to porcine cartilage and could deliver CK to cartilage tissues by sealing cartilage defects. The CK drugs from the CHI-HCA patches were effective in the prevention of cartilage degradation by inhibiting degradative enzymes and apoptotic cell deaths via inhibiting NFkB signaling. Thus, we expect that CK-loaded adhesive patches have enormous potential as drug-loaded sealing materials for cartilage regenerations and the prevention of further cartilage degradation, particularly for the surgical treatment of cartilage repair.

## Supplementary Material

rbad077_Supplementary_Data
