# Supplementary material for: Adhesive ginsenoside compound K patches for cartilage tissue regeneration
Source: Regen Biomater. 2023 Aug 31;10:rbad077. doi: 10.1093/rb/rbad077 (PMC10518074; doi:10.1093/rb/rbad077)
Supplement: rbad077_Supplementary_Data [file rbad077_supplementary_data.docx]

**Supplemental Table 1**. List of Primers used in this study

| Gene symbol | Forward | Reverse |
| --- | --- | --- |
| BDNF | GGTCACAGTCCTAGAGAAAG | CAGCCTTCCTTGGTGTAA |
| BMP6 | AGGACTGGATCATTGCAC | GTTGGTGGCATTCATGTG |
| CCL2 | CTCAGCCAGATGCAGTTA | GTGATCCTCTTGTAGCTCTC |
| CXCL3 | CAGAAGTCATAGCCACTCTC | CTTGCCGCTCTTCAGTAT |
| CXCL2 | GACAGAAGTCATAGCCACTCTC | GCCTTGCCTTTGTTCAGTATC |
| CCND1 | CAGAGGCGGATGAGAACAAG | GAGGGTGGGTTGGAAATGAA |
| DYNLT3 | CAAGCATAGTGGAACAGTCT | CTGGCTGTGTGAAATCCATA |
| FGF2 | TACCGGTCACGGAAATAC | GAAGAAACAGTATGGCCTTC |
| FGF7 | GCGACACACCAGAAGTTA | CTGGGTCCCTTTCACTTT |
| GDNF | CTGACCAGTGACTCCAATATG | GCTTGTTTATCTGGTGACCT |
| GBE1 | CAAGAGCTATACGGACTACC | GTCTCTGATGACCTCCATAC |
| IGFBP4 | CGAACATCCCAACAACAG | CATCTTGCTCCGATCTCTA |
| IGFBP2 | AAGTCAGGCATGAAGGAG | TCCAGACTGAGGTGTTTG |
| IGFBP7 | GTGCTGGTATCTCCTCTAAG | GGGCATCAACCACTGTAA |
| ICAM1 | GTACTGCTGGTCATTGTG | CCTGAGCCTTCTGTAACT |
| UFM1 | GCTAAAGTTTGCAGCAGAAG | ACATTCCCAGCAGTCTGT |
| MMP3 | GGACCAGGGATTAATGGAGATG | TGAGCAGCAACCAGGAATAG |
| MMP10 | GTGGTGTTCCTGATGTTG | ATCCACACTCTGTCTTGG |
| MMP12 | GAAGCGGTACCTCACTTA | AGTCACATCACTCCAGAC |
| MST1 | CTTCCACTACAACATGAGC | CACAGACTCGAGTGGTATAG |
| TMEM87B | GACAAATCAGGACCTTTGG | GCCACTCTATGGTAAACTTC |
| P4HA2 | AAGCTCGCCAAGATTAAGAG | GTAGGCATTCACAGGATGAG |
| PLK3 | GTCTGTTTGCCAAAGTTACC | ACCAAGCAGGAGACATTATC |
| PLXNA3 | CCTTCTCCCATTGCTATTC | GTGAGTCAGAGTGGTATCT |
| TAF13 | CTGAAATGACTCACAAGGC | CTCTAGCAAACTTCCTTGG |
| RAI14 | AAGATTCTTCCGGTCACAG | CCGAGTTGTCAATGTTCTC |
| TSPAN13 | CTTGTTTAGCTCTGAATCGG | GCAGTTCAAATTCCTCTGG |
| TNFRSF11B | GAAACCCTTCCTCCAAAGTA | CAATGTCTTCCTCCTCACTG |
| SLC16A3 | CTGCAGAAGCATTATCCAGA | ATTGAGCATGATGAGGGAAG |
| TNFRSF1B | CTGTAGCATCCTGGCTATTC | TCTGGCTGAGATACGTAGAG |
| VEGFA | CTTTACTGCTGTACCTCCAC | CTGGTAGACATCCATGAACT |
| MMP3 | GGACCAGGGATTAATGGAGATG | TGAGCAGCAACCAGGAATAG |
| ADAMTS4 | GCATTCCATGGTACAGGGTTA | AGTTGACAGGGTTTCGGATG |
| Bax | AGAGGATGATTGCTGACG | AAGTAGAAGAGGGCAACC |
| Casp3 | CAGTGGACTCTGGGATCTATCT | TGACATTCCAGTGCTCTTATGG |
| Casp9 | AGACCTTGGATGGCATTCTG | CAGCCAGGAATCTGCTTGTA |
| Bcl2l2 | CTGAGGCAGAAGGGTTAT | GTCTCAAACTCGTCTCCA |
| AKT1 | CTTCTATGGTGCGGAGAT | ATGAGGTTCTCCAGCTTC |
| Annexin A2 | GAATCATGGTCTCTCGCA | GGTGTCTTGCTGGATGTA |
